# Potential Protective Effects of Ursolic Acid against Gamma Irradiation-Induced Damage Are Mediated through the Modulation of Diverse Inflammatory Mediators

**DOI:** 10.3389/fphar.2017.00352

**Published:** 2017-06-16

**Authors:** Hong Wang, Meng-Kwoon Sim, Weng Keong Loke, Arunachalam Chinnathambi, Sulaiman Ali Alharbi, Feng Ru Tang, Gautam Sethi

**Affiliations:** ^1^Department of Pharmacology, Yong Loo Lin School of Medicine, National University of SingaporeSingapore, Singapore; ^2^Singapore Nuclear Research and Safety Initiative, National University of SingaporeSingapore, Singapore; ^3^Agent Diagnostic and Therapeutic Laboratory, Defence and Environmental Research Institute, DSO National LaboratoriesSingapore, Singapore; ^4^Department of Botany and Microbiology, College of Science, King Saud UniversityRiyadh, Saudi Arabia; ^5^School of Biomedical Sciences, Curtin Health Innovation Research Institute, Curtin University, PerthWA, Australia

**Keywords:** radiation, ursolic acid, NF-κB, TNF-α, NO

## Abstract

This study was aimed to evaluate the possible protective effects of ursolic acid (UA) against gamma radiation induced damage both *in vitro* as well as *in vivo*. It was observed that the exposure to gamma radiation dose- and time-dependently caused a significant decrease in the cell viability, while the treatment of UA attenuated this cytotoxicity. The production of free radicals including reactive oxygen species (ROS) and NO increased significantly post-irradiation and further induced lipid peroxidation and oxidative DNA damage in cells. These deleterious effects could also be effectively blocked by UA treatment. In addition, UA also reversed gamma irradiation induced inflammatory responses, as indicated by the decreased production of TNF-α, IL-6, and IL-1β. NF-κB signaling pathway has been reported to be a key mediator involved in gamma radiation-induced cellular damage. Our results further demonstrated that gamma radiation dose- and time-dependently enhanced NF-κB DNA binding activity, which was significantly attenuated upon UA treatment. The post-irradiation increase in the expression of both phospho-p65, and phospho-IκBα was also blocked by UA. Moreover, the treatment of UA was found to significantly prolong overall survival in mice exposed to whole body gamma irradiation, and reduce the excessive inflammatory responses. Given its radioprotective efficacy as described here, UA as an antioxidant and NF-κB pathway blocker, may function as an important pharmacological agent in protecting against gamma irradiation-induced injury.

## Introduction

Gamma rays constitute an important kind of ionizing radiation with strong penetrating ability. Ionizations induced by gamma radiation may act directly on component molecules of cells or indirectly on water molecules, causing water radiolysis and further generating free radicals (Lee et al., 2012; Wang et al., 2015). Free radicals interact with adjacent molecules, thereby resulting in DNA damage, oxidation, and inflammation (Wang et al., 2015; Fernandez-Viadero et al., 2016; Haase and Prasad, 2016). Free radicals-caused peroxidation of lipids leads to structural and functional damage to cellular membranes. Lipid peroxidation, together with DNA damage, constitutes the key events involved in radiation induced cell death. UA, as a pentacyclic triterpenoid antioxidant, is widely found in various medicinal herbs such as *Rosemarinus officinalis, Eugenia jumbolana, and Ocimum sanctum*, as well as in privet (*Ligustrum*) and hawthorn (*Cratagegi* sp.) fruits (Lee et al., 2012; Valdes et al., 2016; Cargnin and Gnoatto, 2017). These plants have been used to alleviate inflammatory conditions and reduce hypertension since ancient times in several countries (Najid et al., 1992; Ali et al., 2007; Cho and Lee, 2016; Peng et al., 2016). The various studies have shown that UA exhibits its diverse pharmacological effects including its potent anticancer activity against various types of cancer cells (Shanmugam et al., 2013). Several phase I clinical trials have been conducted to evaluate the safety, tolerance and antitumor activity of intravenously administrated UAL. It was found that UAL was tolerable with manageable levels of toxicity, and could potentially improve remission rates in patients (Wang et al., 2013, 2015; Zhu et al., 2013; Qian et al., 2015).

The anticancer and anti-wrinkle effects of UA and its related derivatives as medicinally important natural products have also been clinically tested (Sultana, 2011; Cho and Lee, 2016; Martinez-Abundis et al., 2016). Interestingly, UA was found not only to significantly inhibit tumor growth, but also to enhance the recovery of hematopoietic system post-irradiation in mice (Hsu et al., 1997). It has been reported that UA exhibits potent antioxidant activity (Yin and Chan, 2007), and is also effective in preventing lipid peroxidation in an *in vitro* model (Balanehru and Nagarajan, 1991). So far, very little literature is available regarding the functions of UA in gamma radiation protection. However, the protective effects of UA against damage induced by UV radiation have been demonstrated in human cells. One study showed that UA significantly inhibited the UVA-induced signal transduction pathways in HaCaT human skin cells, and prevented UVA-induced photoaging (Soo Lee et al., 2003). UA was also found to exert a protective effect on UVB-induced lipid peroxidation and oxidative DNA damage in human lymphocytes (Ramachandran and Prasad, 2008). Moreover, UA can also inhibit the activation of transcription factor NF-κB at multiple steps, including its phosphorylation and the ability of p50/p65 heterodimer to bind to DNA (Shishodia et al., 2003; Shanmugam et al., 2011a,b, 2012; Checker et al., 2012).

NF-κB is an important transcription factor that can be activated in response to a variety of inflammatory stimuli, such as toxins, pathogens, and radiation-induced oxidative stress (Pahl, 1999; Raju et al., 2000; Mohan et al., 2007; Sethi et al., 2008, 2012; Sethi and Tergaonkar, 2009; Li and Sethi, 2010; Chai et al., 2015; Li et al., 2015). It acts as a redox sensor and has been reported to participate in the activation of radiation-induced inflammatory cascade, DNA damage and NO production (Ibuki et al., 2003; Manna et al., 2015). The treatment of diverse pharmacological agents, such as Naringin (Manna et al., 2015), Corilagin (Dong et al., 2010), Brazilin (Kang et al., 2016), and Baicalein (Pfalzgraff et al., 2016), have been previously reported to significantly reverse the inflammatory responses and oxidative DNA damage through the modulation of NF-κB signaling pathways. Therefore, we hypothesized that UA, being a potent NF-κB blocker, may also display protective effects against injury induced by gamma radiation. The present study was carried out to investigate the potential role of this triterpene in alleviating radiation-induced damage both *in vitro* and *in vivo*.

## Materials and Methods

### Reagents

Ursolic acid, DMSO, DHE, nuclease P1, alkaline phosphatase, MTT, CelLytic^TM^ mammalian tissue lysis/extraction reagent, sodium dodecyl sulfate (SDS), Tris, glycine, and BSA were obtained from Sigma–Aldrich (St. Louis, MO, United States). DCFDA cellular ROS detection assay kit was purchased from Abcam (Cambridge, MA, United States). DMEM, FBS, and antibiotic-antimycotic mixture were purchased from Invitrogen (Carlsbad, CA, United States). EMEM was obtained from ATCC (Manassas, VA, United States). Griess reagent and genomic DNA purification Kit were purchased from Promega (Madison, WI, United States). DPPP, and EIA kits for 8-OH-dG were obtained from Cayman Chemical Company (Ann Arbor, MI, United States). IL-6, IL-1β, and TNF-α ELISA kits were purchased from R&D system (Minneapolis, MN, United States). Nuclear extraction kit and TransAM^TM^ NF-κB Transcription Factor assay kits were obtained from Active Motif (CA, United States). Antibodies against p65, phospho-specific p65 (Ser 536), phospho-specific IκBα (Ser 32), and β-actin were obtained from Cell Signalling Technology (Beverly, MA, United States). Antibodies against PARP and IκBα, HRP-conjugated goat anti-rabbit and anti-mouse antibodies were purchased from Santa Cruz Biotechnology (Santa Cruz, CA, United States). Bradford reagents, and nitrocellulose membrane, were obtained from Bio-Rad (Hercules, CA, United States) and Western Blotting Detection ECL Reagent was purchased from GE healthcare (Buckinghamshire, United Kingdom).

### Cell Lines and Drug Treatment

HaCaT cells were kindly provided by Dr. Francoise Thierry, Institute of Medical Biology, Agency for Science Technology and Research (A^∗^STAR), Singapore. It is an immortalized human epidermal keratinocyte, which was *in vitro* spontaneously transformed from histologically normal skin. These cells were cultured in DMEM containing 10% FBS and 1% antibitotic-antimycotic solution. BJ human skin fibroblast cells derived from normal foreskin were purchased from ATCC (CRL-2522, Manassas, VA, United States), and cultured in ATCC-formulated EMEM containing 10% FBS and 1% antibitotic-antimycotic solution. All cells were maintained at 37°C in a humidified atmosphere 5% CO_2_. All the cultures were routinely tested and were mycoplasma-free. UA was dissolved in DMSO as a 100 mM stock solution and stored at 4^o^C. It was diluted in cell medium or saline before *in vitro* or *in vivo* administration.

### Irradiation Procedure

Cells were treated with UA at designated concentrations for 16 h, and then subjected to gamma-radiation in the Gamma-irradiator BIOBEAM 8000 (Gamma-Service Medical GmbH, Leipzig, Germany) with indicated doses. DMEM or EMEM group without any drug treatment served as control. The cells were harvested and subjected to different assays at designated time points after irradiation.

### Cell Viability Assay

The cell viability after irradiation and UA treatment was evaluated by MTT method. The assay is based on the cleavage of yellow tetrazolium salt MTT to form purple formazan crystals in metabolically active cells. Briefly, cells were incubated with or without treatment in triplicate in a 96-well plate. Twenty microliter PBS containing 5 mg/mL MTT was added to the plate, and incubated at 37°C for 4 h. After removal of the supernatant, 0.1 mL lysis buffer (50% dimethylformamide plus 20% SDS) was added, incubated at 37°C for 1 h, and then the optical density (OD) was measured at 570 nm by Tecan Safire microplate reader (Thermo Fisher Scientific, Waltham, MA, United States).

### ROS and NO Production Assays

Reactive oxygen species production was assayed by DHE and DCFDA. DHE upon reaction with superoxide anions forms a red fluorescent product 2-hydroxyethidium, which can be monitored at an excitation wavelength of 535 nm and an emission wavelength of 610 nm. A cell-permeable and non-fluorescent probe DCFDA is de-esterified and turns to fluorescent 2′, 7′-dichlorofluorescein (DCF) intracellularly upon oxidation. Florescence DCF can be read at excitation and emission wavelengths of 485 and 535 nm. Cells were treated with 5 μM DHE or 25 μM DCFDA at 37°C for 30 or 45 min in the dark, respectively, and then subjected to the fluorescent reading by Varioskan^TM^ Flash microplate reader (Thermo Scientific, Waltham, MA, United States).

Griess Reagent was used to evaluate the ${\mathrm{NO}}_{2}^{–}$ concentration. Fifty microliter nitrite standard or sample was incubated with 50 μl Sulfanilamide Solution and 50 μl NED Solution at room temperature for 5–10 min in the dark, and then a purple/magenta color formed. The absorbance was measured at 520 nm wavelength within 30 min in a plate reader.

### Lipid Peroxidation Assay

Lipid peroxidation was assayed by DPPP, a probe that stoichiometrically reacts with hydroperoxides to yield the fluorescent DPPP-O, which can be measured using excitation wavelength at 351 nm and emission wavelength at 380 nm, respectively. Cells were incubated with 50 μM DPPP at 37°C for 60 min in the dark, and then subjected to the fluorescent measurement using Varioskan^TM^ Flash microplate reader.

### Assay for DNA Oxidative Damage

Cells were harvested by trypsinization, incubated with proteinase K, RNase A, and lysis/binding buffer at 55°C for 10 min. The lysate was added into the spin column, centrifuged, washed, and eluted. The concentration of purified DNA was measured.

DNA oxidative damage was evaluated by 8-OH-dG EIA kit according to the manufacturer’s instructions. Gamma radiation induced DNA oxidative damage initiates the repair process which releases multiple oxidized guanine species. The Cayman’s assay is developed to measure the oxidized guanine species, including 8-OH-dG. Briefly, the above-purified DNA was digested by nuclease P1, incubated with alkaline phosphatase at 37°C for 30 min, boiled for 10 min and placed on ice. The plate was set up by the addition of 50 μl standard or sample, 50 μl Tracer, and 50 μl antibodies and then incubated 18 h at 4°C. The plate was washed, and added 200 μl Ellman’s reagent. Optimum development was obtained by shaking the plate in the dark for 90–120 min. The absorbance reading at a wavelength of 420 nm was recorded and used to calculate the 8-OH-dG concentration based upon the standard curve.

### Enzyme-Linked Immunosorbent Assay

The levels of TNF-α, IL-6, and IL-1β in cell lysate were evaluated using ELISA. Hundred microliter standard or sample solutions were added into a capture antibodies coated 96-well plate, which was then blocked by 300 μl Reagent Diluent. After 2 h incubation at room temperature, the plate was washed, and incubated sequentially with detection antibodies, streptavidin-HRP, substrate and stop solution. The OD of each well was measured at 450 nm, and the concentrations of inflammatory molecules were calculated based upon the standard curve.

### Nuclear and Cytoplasmic Fraction Extraction

The cells were washed with PBS containing phosphatase inhibitors, removed by gently scraping, and centrifuged at 500 rpm for 5 min. The cell pellet was re-suspended in 1× hypotonic buffer by pipetting up and down several times. After 25 μl detergent was added, the cells were centrifuged for 30 s at 14,000 × *g*, and then the supernatant (cytoplasmic fraction) was collected and stored at -80°C. The nuclear pellet was re-suspended in complete lysis buffer, vortexed, kept on ice for 30 min, and centrifuged for 10 min at 14,000 × g. The supernatant (nuclear fraction) was collected and stored at -80°C.

### NF-κB DNA Binding Activity Assay

To determine NF-κB activity, DNA binding assay was performed using TransAM^TM^ NF-κB transcription factor assay kit according to the manufacturer’s instruction. Briefly, 30 μl complete binding buffer was added to each well in the plate, followed by 20 μl sample diluted in complete lysis buffer. Twenty microliter complete lysis buffer replaced sample solution in blank wells. The plate was incubated for 1 h at room temperature with mild agitation (100 rpm), and washed three times. Hundred microliter diluted NF-κB antibody was added, and thereafter the plate was incubated for 1 h at room temperature without agitation. After washing, HRP-conjugated antibody, developing solution, and stop solution were added sequentially. The absorbance at 450 nm was read within 5 min.

### Western Blot Analysis

The western blot analysis was employed to test the protein expression of p65, phospho-p65, IκBα, phospho-IκBα, PARP, and β-actin. The cells were lysed in lysis buffer, and centrifuged for 15 min at 14,000 × *g* to pellet the cellular debris. The protein-containing supernatant was collected, and the protein concentration was evaluated by Bradford method. The cell lysates were resolved by electrophoresis on a 10% SDS-PAGE gel. The proteins were transferred to a nitrocellulose membrane, blocked by 5% non-fat milk, and probed with primary antibodies against p65, phospho-p65, phospho-IκBα, IκBα, PARP, and β-actin overnight at 4°C. The membrane was washed, incubated with HRP-conjugated secondary antibodies for 1 h, and examined by ECL method. The image was captured and analyzed by Bio-Rad Gel Doc system. Band density was measured and normalized to that to the respective loading control. Fold change values relative to non-irradiated groups from independent experiments were calculated and expressed in densitometric diagram.

### *In Vivo* Studies and Irradiation Procedure

Seven to eight week Balb/c female mice were purchased from InVivos, Singapore, and kept with free access to water and food in Comparative Medicine Facility, National University of Singapore. All procedures for animal studies were approved and performed in accordance with the protocols and guidelines of the IACUC, National University of Singapore (IACUC Number: 081/12). Four groups of mice were employed in this study. Non-irradiated control and UA only groups received the ip injection of 0.1% DMSO or UA (25 mg/kg bw) for 30 days without exposure to gamma irradiation. 6.4 Gy and UA+ 6.4Gy group mice received the ip injection of 0.1% DMSO or UA (25 mg/kg bw) for 14 days, followed by exposure to 6.4 Gy gamma irradiation at day 15 and same treatment till day 30. The weight was measured and the general health of each animal was monitored daily. The survival of the animals was recorded daily, and mice were euthanized for blood and colon collection at day 30.

### Blood Count Analysis

The blood from each mouse was collected in EDTA tubes by heart puncture. The animals were anaesthetized in a 3% isoflurane chamber. The fur on chest was trimmed off, and heart puncture was done. The blood was drawn and transferred to EDTA tube. The complete blood count was performed using the Abbott Cell-Dyn 3700 Hematology Analyzer. The numbers of lymphocyte, monocyte, white blood cell, and platelet were recorded and compared.

### ELISA Assay in Mouse Plasma and Western Blot Analysis in Mouse Colon Tissues

The blood was centrifuged at 2,000 × *g* for 10 min in a refrigerated centrifuge to isolate plasma. The content of IL-6 and TNF-α in the plasma was assayed using ELISA kits as described above. The colon tissues from mice were collected at day 30, washed by PBS, and homogenized in CelLytic lysis buffer. After centrifugation for 15 min at 14,000 × g, the protein-containing supernatant was collected. The expression of p65 and phospho-p65 was evaluated by western blot as described above.

### Statistical Analysis

All normally distributed data were expressed as mean ± SEM. The between-group comparisons were analyzed by One-Way ANOVA followed by Tukey’s *post hoc* test. The survival analysis of four groups of mice was examined by Kaplan–Meier method. A *p*-value < 0.05 was considered as statistically significant.

## Results

### UA Dose-Dependently Reversed the Decrease in Cell Viability Induced by Gamma Irradiation

**FIGURE 1:**
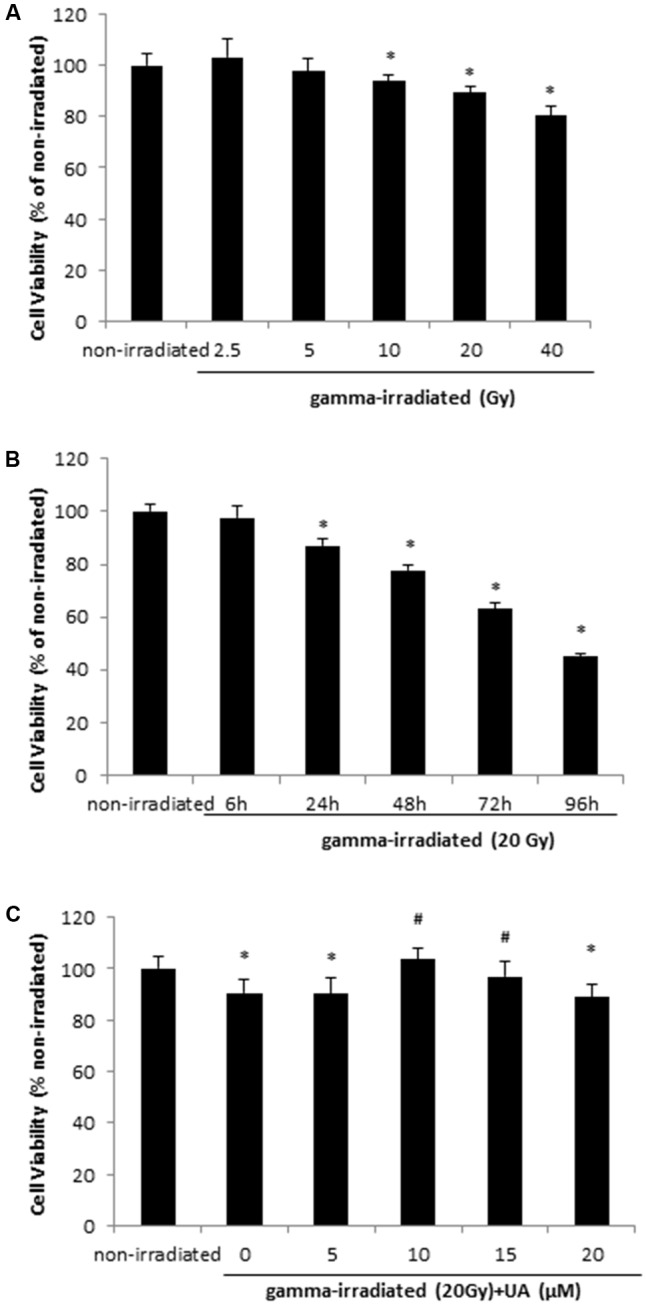
HaCaT cell viability after gamma radiation at various doses **(A)**, various time points **(B)**, and various UA concentration treatment **(C)**. **(A)** HaCaT cells without any treatment were exposed to various doses of gamma radiation from 2.5 to 40 Gy. The cell viability was evaluated after 24 h by MTT. **(B)** HaCaT cells without any treatment were exposed to 20Gy gamma radiation. The cell viability was evaluated by MTT 6 to 96 h post-exposure. **(C)** HaCaT cells were treated with UA at 0, 5, 10, 15, and 20 μM 16 h before exposure to 20 Gy gamma radiation. DMEM group without any drug treatment and gamma radiation exposure served as control. The cell viability was evaluated by MTT 24 h post-exposure. All data were expressed as mean ± SEM. Comparisons between groups were analyzed by One-Way ANOVA followed by Tukey’s *post hoc* test. ^∗^*p* < 0.05 vs. non-irradiated. #*p* < 0.05 vs. irradiated and no drug treatment.

HaCaT cells were exposed to 2.5, 5, 10, 20, and 40 Gy gamma radiation and after 24 h, cell viability significantly decreased at the doses of 10 to 40 Gy when compared with non-irradiated group (**Figure 1A**), indicating gamma irradiation can dose-dependently decrease cell viability in HaCaT cells. In addition, cell viability was measured at 6, 24, 48, 72, and 96 h after exposure of the cells to 20 Gy gamma radiation. It was found that cell viability significantly decreased from 24 to 96 h, thereby suggesting the time-dependent effects of gamma-radiation on cell survival (**Figure 1B**). Therefore, 24 h incubation post 20 Gy gamma radiation exposure was used for following experiments. **Figure 1C** shows that 10 and 15 μM UA treatment significantly reversed the decrease in cell viability induced by irradiation with the peak effects observed at 10 μM. 5 and 20 μM UA treatment showed no significant effects on cell viability, thereby suggesting that 10 and 15 μM are the most effective doses.

A similar trend was observed in BJ human skin fibroblast cells. Exposure to 20 Gy gamma radiation significantly decreased the cell viability after 24 h, while 10 μM UA treatment reversed this decrease (**Supplementary Figure S1**).

### UA Reversed the Increase in ROS and NO Production Induced by Gamma Irradiation

**FIGURE 2:**
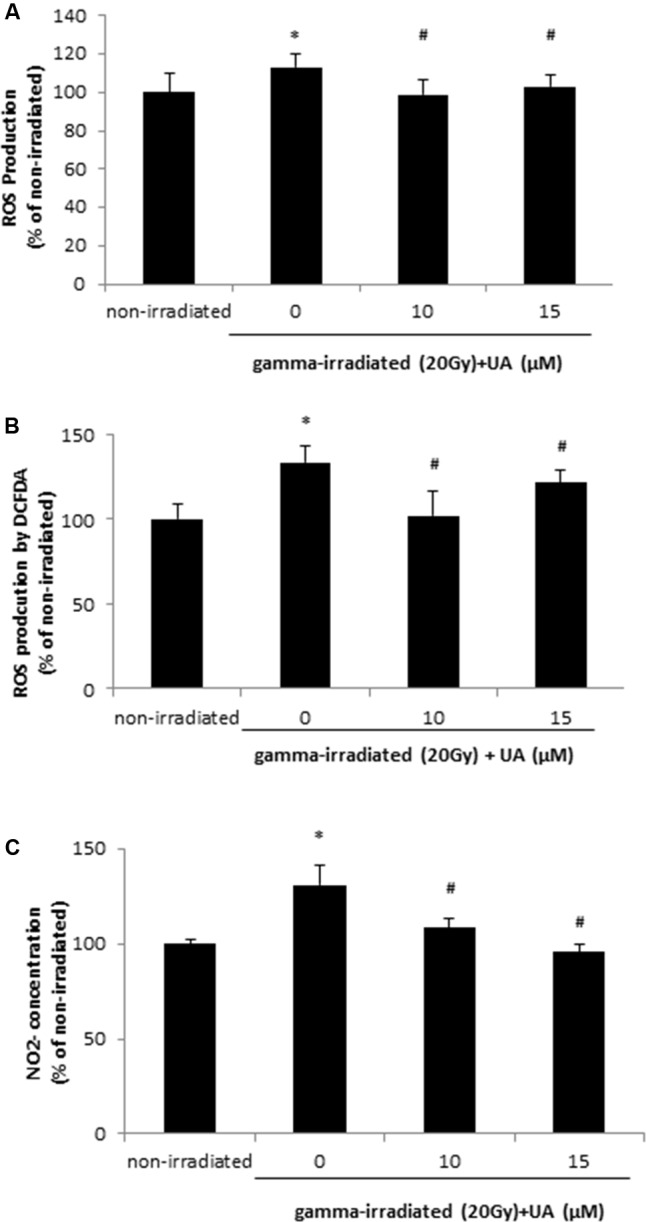
Effects of UA on free radical production in HaCaT cells after gamma radiation. HaCaT cells were treated with UA at 0, 10, and 15 μM 16 h before exposure to 20 Gy gamma radiation. DMEM group without any drug treatment and gamma radiation exposure served as control. ROS production was assayed by 5 μM DHE **(A)** or 25 μM DCFDA **(B)**, and then subjected to the fluorescent reading. **(C)** ${\mathrm{NO}}_{2}^{–}$ concentration was evaluated by Griess Reagent. The absorbance was measured at 520 nm wavelength. All data were expressed as mean ± SEM. Comparisons between groups were analyzed by One-Way ANOVA followed by Tukey’s *post hoc* test. ^∗^*p* < 0.05 vs. non-irradiated. #*p* < 0.05 vs. irradiated and no drug treatment.

DHA and DCFDA assays demonstrated that 20 Gy gamma radiation induced ROS production at 24 h in HaCaT cells, while treatment with 10 and 15 μM UA significantly blocked this effect and decreased the ROS production (**Figures 2A,B**). NO level in HaCaT cells was also increased 24 h after 20 Gy gamma radiation. This increase could be reversed by the treatment of 10 and 15 μM UA (**Figure 2C**), thereby indicative of the potent anti-oxidative effects of UA in HaCaT cells post gamma-irradiation. ROS production was increased after 20Gy gamma radiation in BJ human skin fibroblast cells, and UA treatment significantly attenuated the increase (**Supplementary Figure S2**).

### UA Attenuated the Increase in Lipid Peroxidation and DNA Oxidative Damage Induced by Gamma Irradiation

**FIGURE 3:**
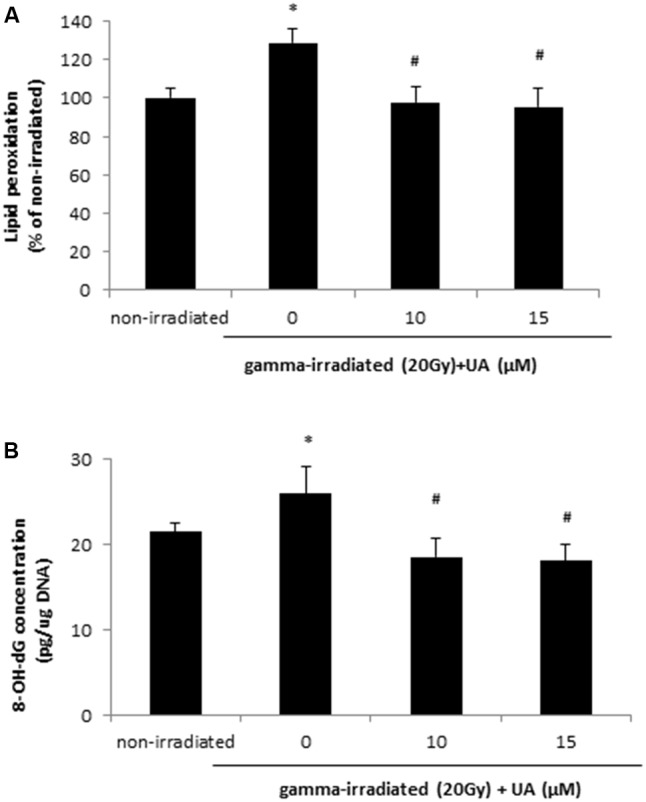
Effects of UA on lipid peroxidation **(A)** and oxidative DNA damage **(B)** in HaCaT cells after gamma radiation. HaCaT cells were treated with UA at 0, 10, and 15 μM 16 h before exposure to 20 Gy gamma radiation. DMEM group without any drug treatment and gamma radiation exposure served as control. **(A)** Lipid peroxidation in HaCaT cells was assayed by 50 μM DPPP. Fluorescence was read using excitation and emission wavelengths of 351 and 380 nm, respectively. **(B)** Oxidative DNA damage was evaluated by 8-OH-dG EIA kit. The absorbance at a wavelength of 420 nm was recorded and used to calculate the 8-OH-dG concentration based upon the standard curve. All data were expressed as mean ± SEM. Comparisons between groups were analyzed by One-Way ANOVA followed by Tukey’s *post hoc* test. ^∗^*p* < 0.05 vs. non-irradiated. #*p* < 0.05 vs. irradiated and no drug treatment.

Free radical can cause rapid lipid peroxidation, which was increased in HaCaT cells at 24 h after 20 Gy gamma radiation (**Figure 3A**). The treatment of 10 and 15 μM UA significantly blocked the increase in lipid peroxidation levels induced by 20Gy gamma radiation (**Figure 3A**). Exposure to 20 Gy gamma radiation also caused DNA oxidative damage in HaCaT cells at 24 h, while the treatment of 10 and 15 μM UA significantly blocked this damage (**Figure 3B**), thereby suggesting that the protective effects of UA against gamma radiation induced injury in HaCaT cells. Interestingly, in BJ human skin fibroblast cells, 10 μM UA treatment significantly reversed the increase in lipid peroxidation and DNA oxidative damage induced by 20 Gy gamma radiation (**Supplementary Figure S3**).

### UA Reversed Gamma Irradiation-Induced Increase in the Production of Pro-inflammation Cytokines

**FIGURE 4:**
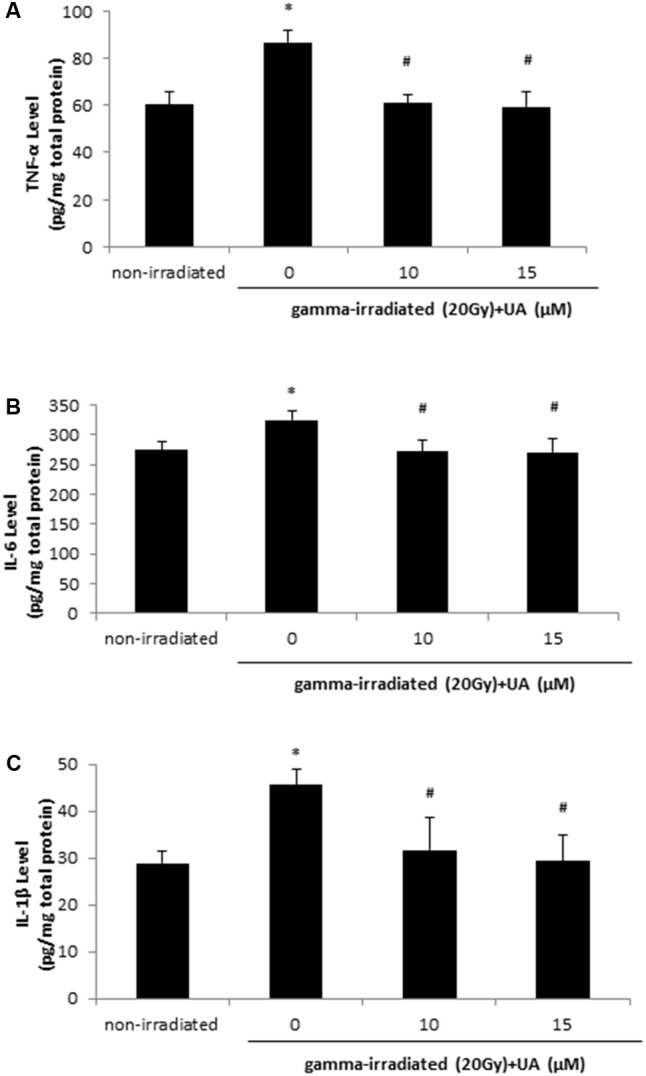
Effects of UA on the levels of inflammatory cytokines: TNF-a, IL-6, and IL-1β. HaCaT cells were treated with UA at 0, 10, and 15 μM 16 h before exposure to 20 Gy gamma radiation. DMEM group without any drug treatment and gamma radiation exposure served as control. The levels of TNF-α **(A)**, IL-6 **(B)**, and IL-1β **(C)** in cell lysate were evaluated using ELISA. All data were expressed as mean ± SEM. Comparisons between groups were analyzed by One-Way ANOVA followed by Tukey’s *post hoc* test. ^∗^*p* < 0.05 vs. non-irradiated. #*p* < 0.05 vs. irradiated and no drug treatment.

The levels of pro-inflammation cytokines TNF-α, IL-6, and IL-1β were increased at 24 h after 20 Gy gamma radiation in HaCaT cells when compared with non-irradiated group (**Figures 4A–C**). The treatment of 10 and 15 μM UA significantly lowered the levels of TNF-α, IL-6, and IL-1β, which was indicative of the potential anti-inflammatory effects of UA in HaCaT cells post gamma-irradiation.

### UA Inhibited NF-κB Activation Induced by Gamma Radiation

**FIGURE 5:**
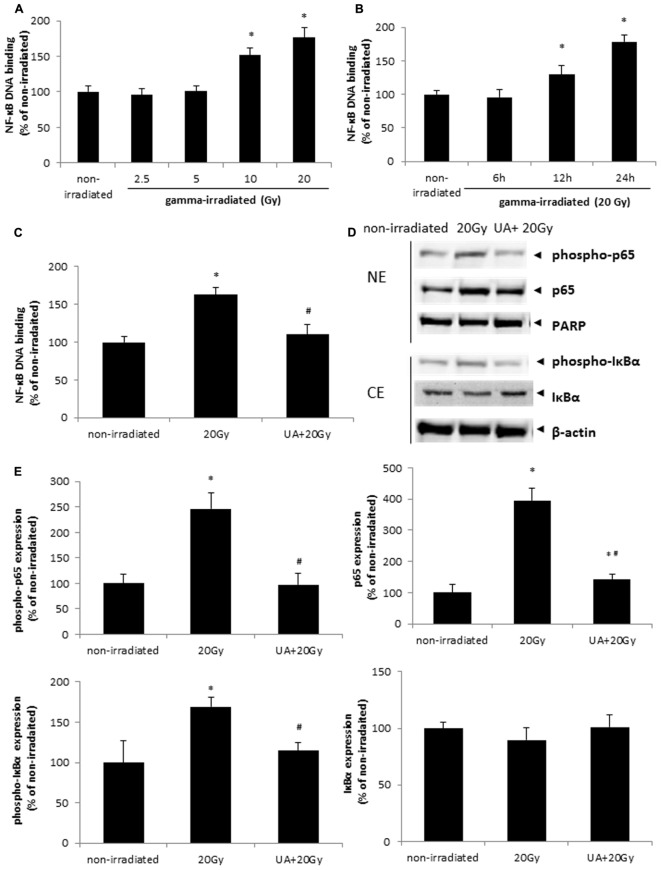
Effects of UA on gamma irradiation-induced NF-κB activation. **(A)** HaCaT cells without any drug treatment were exposed to various doses of gamma radiation from 2.5 to 20 Gy. NF-κB DNA binding assay was performed 24 h post-exposure. **(B)** HaCaT cells without any drug treatment were exposed to 20 Gy gamma radiation. NF-κB DNA binding assay was performed 6, 12, and 24 h post-exposure. **(C)** HaCaT cells with or without 10 μM UA treatment were exposed to 20 Gy gamma radiation. DMEM group without any drug treatment and gamma radiation exposure served as control. NF-κB DNA binding assay was performed 24 h post gamma-radiation. **(D,E)** Phosphorylation and translocation of NF-κB were evaluated by western blot analysis. HaCaT cells with or without 10 μM UA treatment were exposed to 20 Gy gamma radiation. DMEM group without any drug treatment and gamma radiation exposure served as control. Representative image of western blotting **(D)** and densitometric analysis **(E)** of the expression of p65, phospho-p65, PARP, phospho-IκBα, IκBα, and β-actin. NE, nuclear extract; CE, cytoplasmic extract. Data were expressed as mean ± SEM. Comparisons between groups were analyzed by One-Way ANOVA followed by Tukey’s *post hoc* test. ^∗^*p* < 0.05 vs. non-irradiated. #*p* < 0.05 vs. irradiated and no drug treatment.

It was found that NF-κB DNA binding activity was increased at 24 h after 10 and 20 Gy gamma radiation (**Figure 5A**). 20 Gy gamma radiation induced a significant increase in NF-κB DNA binding activity in HaCaT cells from 12 to 24 h (**Figure 5B**). These data indicated that gamma irradiation could promote the activation of NF-κB pathway in a dose- and time-dependent manner. However, 10 μM UA treatment significantly blocked the increase of NF-κB DNA binding activity induced by 20 Gy gamma radiation (**Figure 5C**). Western blot result showed the higher expression of p65, phospho-p65, and phospho-IκBα after 20Gy gamma radiation in HaCaT cells, while 10 μM UA treatment substantially attenuated the expression of these inflammatory proteins (**Figures 5D,E**). These results suggested that UA could act as a potent NF-κB inhibitor and block both the phosphorylation as well as the nuclear translocation of p65.

NF-κB DNA binding activity and the expression of p65, phospho-p65, and phospho-IκBα were all increased after 20Gy gamma radiation in BJ human skin fibroblast cells, while 10 μM UA treatment decreased these enhancements (**Supplementary Figure S4**).

### UA Exposure Significantly Improved the Survival of Mice Following Exposure to Gamma Radiation

**FIGURE 6:**
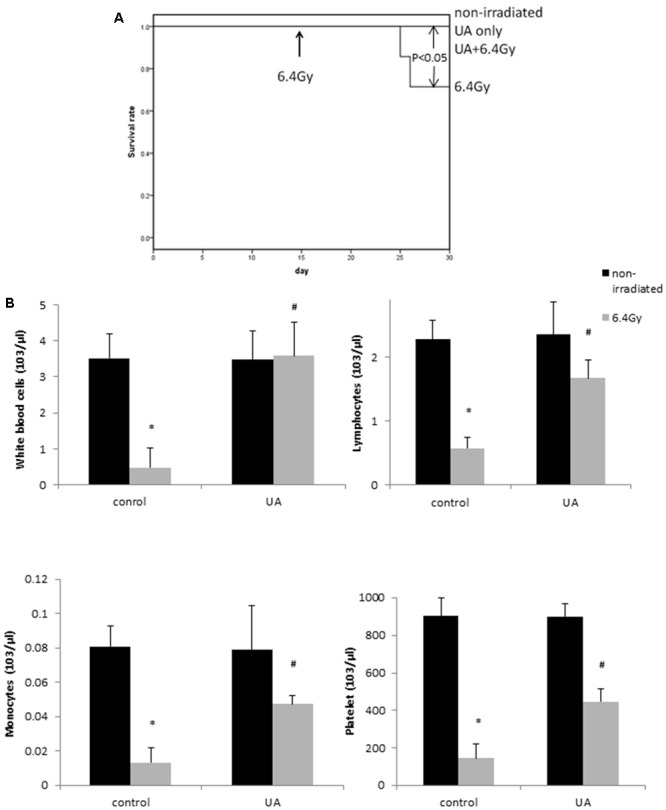
Effects of UA treatment on the survival rate **(A)** and hematopoietic level **(B)** in gamma irradiated mice. Four groups of mice: non-irradiated control and UA only groups received ip injection of 0.1% DMSO or UA (25 mg/kg bw) for 30 days without exposure to gamma irradiation. 6.4 Gy and UA+ 6.4 Gy group mice received ip injection of 0.1% DMSO or UA (25 mg/kg bw) for 14 days, followed by exposure to 6.4 Gy gamma irradiation at day 15 and same treatment till day 30. **(A)** Kaplan–Meier curve of the effects of UA on the survival rate of mice. **(B)** The number of white blood cells, lymphocytes, monocytes, and platelet. Data were expressed as mean ± SEM. The comparisons between groups were analyzed by One-Way ANOVA followed by Tukey’s *post hoc* test. ^∗^*p* < 0.05 vs. non-irradiated and no drug treatment group. #*p* < 0.05 vs. irradiated and no drug treatment group.

Non-irradiated control and non-irradiated UA group mice showed 100% survival rate during 30 days. After gamma-irradiation at day 15, 6.4Gy group mice displayed 20% mortality at day 30, while the mice with UA treatment maintained 100% survival rate (**Figure 6A**). The overall *p*-value < 0.05 clearly indicates that UA treatment could significantly protect the mice against gamma irradiation induced mortality.

### Protective Effects of UA Administration Against Gamma Irradiation Induced Myelosuppression in Mice

The mice exposed to 6.4 Gy gamma irradiation developed myelosuppression: leucopenia, lymphocytopenia, monocytopenia, and thrombocytopenia, as indicated by the reduced levels of white blood cells, lymphocytes, monocytes, and platelets (**Figure 6B**). However, the treatment of UA in mice for 30 days significantly increased the counts of hematopoietic cells. UA treatment alone without gamma irradiation did not show any effects on the expression levels of white blood cells, lymphocytes, monocytes, and platelet. These results indicated that UA could improve the syndromes of myelosuppression at the hematopoietic level.

### UA Treatment Showed Anti-inflammatory Effects in Mice Exposed to Gamma Irradiation

**FIGURE 7:**
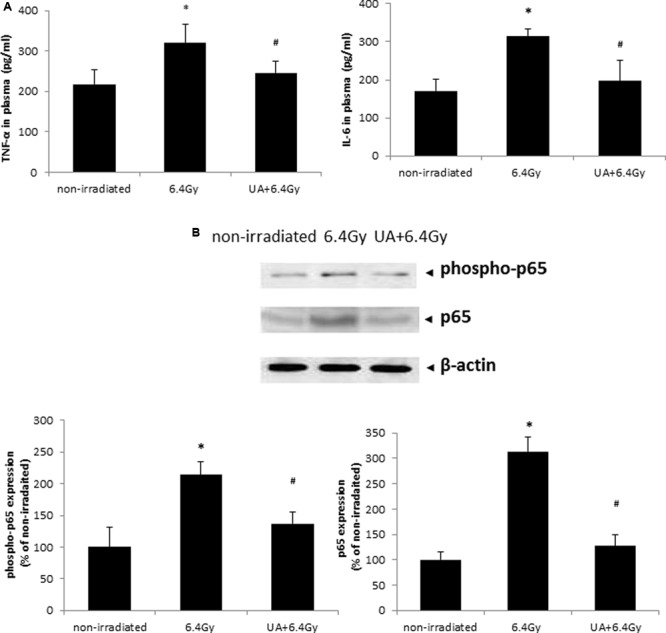
Effects of UA treatment on the expression of inflammatory cytokines **(A)** and NF-κB activation **(B)**. Non-irradiated control did not receive any drug treatment or gamma radiation. 6.4 Gy group mice only exposed to 6.4 Gy gamma radiation without any drug treatment. UA+6.4 Gy group mice received ip injection of UA (25 mg/kg bw) for 14 days, followed by exposure to 6.4 Gy gamma irradiation at day 15 and same treatment till day 30. **(A)** The expression level of inflammatory cytokines TNF-α and IL-6 was evaluated in plasma by ELISA. **(B)** Expression of p65, phospho-p65, and β-actin was evaluated by western blot in colon tissue of mice. The image was captured and analyzed by Bio-Rad Gel Doc system. The results were calculated and expressed as fold change relative to control. Data were expressed as mean ± SEM. Comparisons between groups were analyzed by One-Way ANOVA followed by Tukey’s *post hoc* test. ^∗^*p* < 0.05 vs non-irradiated. #*p* < 0.05 vs irradiated and no drug treatment.

The plasma level of pro-inflammatory cytokines IL-6 and TNF-α was significantly elevated after 6.4 Gy gamma-irradiation as compared to that obtained from the non-irradiated mice (**Figure 7A**). UA treatment showed significant anti-inflammatory effects as indicated by downregulating the expression levels of both IL-6 and TNF-α after exposure to gamma-irradiation.

### UA Inhibited Gamma Irradiation-Induced NF-kB Pathway Activation in Tissues Harvested from Mice

The expression levels of both phospho-p65 and p65 were increased in the colon of mice after 6.4 Gy gamma irradiation, which was indicative of the activation of NF-κB pathway. This increase in NF-κB activation was inhibited upon UA treatment (**Figure 7B**). These results demonstrated the function of UA as a NF-κB inhibitor in blocking the phosphorylation and translocation of NF-κB.

## Discussion

A sustained use of nuclear energy in modern world has simultaneously increased the probability of exposure to radiation in human beings (Thompson, 2001; Fesenko et al., 2017; Ikenoue et al., 2017), and hence development of safe and effective radioprotectors has become an expeditious issue. Several synthetic compounds, such as quercetin, lipoic acid, deoxyspergualin, 2- mercaptopropionylglycine, cysteamine have been screened for their ability for radioprotection (Goyal and Dev, 1983; Nemoto et al., 1995; Hensley et al., 1999; Selim et al., 2010; Das et al., 2014). However, the practical applications of these synthetic radioprotectors are quite limited due to their high systemic toxicity at their optimum protective dose (Hosseinimehr, 2007; Szejk et al., 2016). Thus, such safety concerns demand the search for less or non-toxic compounds from natural sources with pharmacological properties (Jagetia, 2007; Sandeep and Nair, 2011; Kuntic et al., 2013).

A number of medicinal plants have been evaluated for their radioprotective effects. For example, *Podophyllum hexandrum*, also known as Himalayan Mayapple, exerts its functions in radioprotection in lethally irradiated mice (Lata et al., 2009; Sankhwar et al., 2011). The extract of this plant contains several active components that have been shown to have antioxidant potential, ability to inhibit the production of nitric oxide synthase and upregulate DNA repair proteins (Dutta et al., 2012; Saini et al., 2013). *Acorus calamu*s, also called sweet flag or calamus, is a tall perennial herb belonging to Acoraceae family in the genus Acorus. Its extract was found to dose-dependently scavenge free radicals, prevent peroxidation of membrane lipids, protect DNA breaks and enhance the DNA repair process after gamma irradiation in an *in vitro* study using mouse liver homogenate (Sandeep and Nair, 2010). The activity of *Acorus calamus* extract was also tested in mice exposed to whole body lethal and sub-lethal doses of gamma-irradiation. The treatment of this extract significantly increased the survival rate and the activities of antioxidant enzymes SOD, catalase and GPx, and decreased GSH levels and the formation of malondialdehyde (Sandeep and Nair, 2012). These studies highlight that *Acorus calamus* extract may be used as a natural protective agent for radiation-induced injuries. However, the combination of several bio-active components in these plant extracts often makes it difficult to investigate the underlying molecular and biochemical mechanisms contributing to their radioprotective effects.

In this study, we employed UA, a triterpene, which exists in various medicinal herbs and fruits, and explored its potential protective efficacy against gamma radiation-induced cell and tissue injury. Triterpenes are a class of chemical compounds composed of three terpene units, which can be divided based on the number of rings, and in general, pentacyclic structures (5 rings) tend to be dominant. UA possesses a pentacyclic motif with a hydroxyl group at the C-3 position and a carboxylic group at the C-17 position. Although no information on the structure-activity relationships of UA in radioprotection has been published so far, several studies have revealed the important roles of both the hydroxyl group and the carboxylic group in the activities of UA in hepatoprotection (Liu et al., 1994), antiosteoclastogenesis (Tan et al., 2015), and glucosidase inhibition (Wu et al., 2015). UA has been found to be quite safe for pharmacological applications (Cho and Lee, 2016), and our preliminary study also showed that this triterpene exhibited no adverse effects in mice up to a concentration of 100 mg/kg bw daily administered through ip injection for 30 days (data not shown). So far, very little evidence has been published on the ability of UA to offer protection against gamma radiation-induced injury, although its functions in modulating various key hallmarks of cancer cells are well recognized. We observed that the treatment of UA attenuated the gamma radiation-induced cytotoxicity, free radical production, lipid peroxidation generation, oxidative DNA damage, and inflammatory response in human keratinocytes and fibroblasts. Moreover, UA was also found to act as a potent NF-κB inhibitor, and block the gamma radiation induced increase in NF-κB DNA binding activity, as well as its phosphorylation and nuclear translocation.

Human keratinocytes (HaCaT cells) have been widely employed to study UV-induced skin injury, as the skin is the organ susceptible to radiation damage. When skin is exposed to a high-dose radiation, complex pathophysiological reactions, such as severe inflammation, cell death, and tissue destruction occur. This is called the radiation burn or cutaneous radiation syndrome. For example, three patients exposed to high-dose irradiation have been reported in Tokaimura criticality nuclear accident (Hirama et al., 2003). The patients suffered severe skin lesions, and later gastrointestinal bleeding. Our results showed that gamma radiation dose-dependently induced cell death in HaCaT cells within dose range from 10 to 40 Gy, while no significant decrease in cell viability was observed after exposure to dose below 5 Gy. This is consistent with previous reports that 6 Gy or lower radiation doses are not cytotoxic in HaCaT (Gault et al., 2005; Tremezaygues et al., 2010), but X-ray radiation at 20 Gy can decrease cell viability and increase ROS production (Gu et al., 2013).

Reactive oxygen species and NO are both free radicals, which can be generated after exposure to gamma radiation (Wang et al., 2015). Our results also showed that 20 Gy gamma radiation significantly enhanced ROS production and NO release, which further caused an increase in lipid peroxidation and oxidative DNA damage in the cells. Both are the critical events involved in radiation induced cell death. The initiation of robust inflammatory response is also a severe consequent following exposure to gamma radiation, which is characterized by the production of pro-inflammatory cytokines, such as TNF-a, IL-6, and IL-1β (El-Desouky et al., 2016; Ismail et al., 2016). The increased production of these diverse cytokines was observed in our study after exposure to gamma radiation. Inflammation, together with lipid peroxidation and oxidative DNA damage, all compose free radical chain reaction mechanisms that contribute to the various deleterious effects of radiation exposure.

Ursolic acid, as an antioxidant and anti-inflammatory agent, was found to reverse gamma radiation induced cell death at concentrations of 10 and 15 μM, but not 20 μM, showing a biphasic dose-response relationship. The classic dose-response curve of drugs is considered to be sigmoidal, in terms of a drug being ineffective at low concentrations, moderately effective at intermediate concentrations, reaching and retaining at a maximum level of efficacy at high concentrations. However, extensive evidence suggests many drugs and reagents, e.g., anti-angiogenic agents, preferentially exhibit biphasic dose-response models (Reynolds, 2010; Owen et al., 2014). Unlike standard sigmoidal curves, biphasic concentration-response models suggest more complex biological effects of drugs through multiple binding sites or multiple targets. UA has been demonstrated to inhibit angiogenesis and cell growth in different types of cancer through multiple signaling pathways (Shanmugam et al., 2011b; Saraswati et al., 2013; Jin et al., 2016), which might explain its dose-response model in our study, although the exact underlying mechanisms remain to be elucidated.

The radioprotective effect of UA was observed to be achieved through the inhibition on ROS and NO production, and further on the free radical cascades. Our results are in agreement with a previous study from Ramachandran and Prasad (2008), who reported the protective effects of UA against cytotoxicity, lipid peroxidation and DNA damage induced by UV-B radiation in human lymphocytes (Ramachandran and Prasad, 2008). Another report also demonstrated that UA inhibited UVA-radiation-induced oxidative damage in human keratinocytes (Soo Lee et al., 2003). Although UA has been reported to exhibit anti-inflammatory (Peng et al., 2016), anti-carcinogenic (Yin et al., 2016), anti-hypertensive (Somova et al., 2003), hepatoprotective (Saravanan et al., 2006), and neuroprotective (Lu et al., 2007) activities, our study is the first to explore the protective effects of UA on gamma radiation induced injury in both keratinocytes and fibroblasts.

Gamma irradiation has been known to activate NF-κB signaling pathway by diverse mechanisms (Yu et al., 2017). The modulation of NF-κB contributes to the survival of the irradiated cells (Manna et al., 2015). Our results demonstrated that exposure to the gamma radiation dose- and time-dependently increased NF-κB DNA binding activities in HaCaT cells, and UA treatment significantly suppressed this increase. The phosphorylation and translocation of NF-κB were also increased after exposure to gamma radiation, which were blocked upon the treatment of UA. Our observations could be supported by a recent study from Manna et al. (2015), who showed that naringin inhibited gamma radiation-caused inflammation and oxidative DNA damage by controlling p53 and NF-κB signaling pathways in murine splenocytes (Manna et al., 2015). In another report, Lee et al. (2014) showed that UV light exposure produced a loss of proliferation and an activation of NF-κB in the skin melanoma cells, while pre-treatment with UA significantly reduced the amount of phosphorylated NF-κB at 24 h post-exposure (Lee et al., 2014). These pieces of evidence support our hypothesis that the protective effects of UA against gamma radiation induced damage through the inhibition of oxidative stress cascade including ROS production, lipid peroxidation, DNA damage, and NF-kB activation.

The protection of UA against gamma radiation induced injury in animals was also examined in this study. Gamma radiation induced oxidative stress cascade has been previously reported in animal models (Das et al., 2014; Khan et al., 2015). The animal death was observed in mice after exposure to gamma radiation with ^137^Cs (Kindekov et al., 2014) and ^60^Co as sources (Hu et al., 2013; Das et al., 2014; Mortazavi et al., 2015), which was consistent with our result that exposure to 6.4 Gy ^137^Cs source radiation induced 20% animal death at 15 day post-radiation. The 30-day treatment of UA significantly increased the survival rate in mice exposed to whole body gamma irradiation. One possible underlying mechanism of radioprotection caused by UA could be through minimizing the damage on the hematopoietic system of the mice. Hematopoietic syndrome is considered as a major cause of death in mice after total body irradiation, and mostly occurs within 30 days after exposure (Williams et al., 2010; Booth et al., 2012; Rosen et al., 2014). In the present study, the levels of white blood cell, lymphocyte, monocyte, and platelet were decreased in gamma-irradiated mice, which is suggestive of leucopenia, lymphocytopenia, monocytopenia, and thrombocytopenia in these mice. Treatment with UA for 30 days significantly attenuated hematopoietic syndrome, as indicated by the increased counts of white blood cells, lymphocytes, monocytes and platelets as compared to the irradiated non-drug treated mice. Our results are in agreement with a previous observation that UA has the ability to decrease the radiation caused damage in the hematopoietic tissues as indicated by an increase in the number of leukocytes compared to the levels in the radiated mice (Hsu et al., 1997).

Another possible mechanism underlying radioprotection of UA was its ability to reduce excessive inflammatory responses in mice exposed to gamma radiation. We found that the circulating levels of two inflammatory cytokines, IL-6 and TNF-α, were enhanced in surviving mice exposed to gamma irradiation, and UA treatment significantly attenuated the levels. Our findings are congruent with another report by Das et al. (2014), who showed higher level of circulating TNF-α and IL-6 in mice after exposure to the gamma radiation, and that the treatment of an antioxidant ferulic acid can attenuate the inflammation of gamma irradiated mice through reducing the levels of these two cytokines (Das et al., 2014). Interestingly, another study found that the gamma radiation exposure enhanced TNF-α and IL-6 expression and lipid peroxidation in mice, accompanied by the activation of NF-κB (Sinha et al., 2012). Our western blot analysis also revealed that the enhanced expression of NF-κB (p65) and the phosphorylated NF-κB (phospho-p65) in mice after gamma radiation exposure. Gamma radiation has been found to be closely associated with the generation of ROS, and thus can activate redox-sensitive transcription factor NF-κB (Brach et al., 1991). NF-κB is further responsible for the inflammatory responses via mediating the expression of inflammatory cytokines, for example, TNF-α and IL-6. UA treatment suppressed the enhancement of p65 and phospho-p65 expression induced by gamma irradiation in our study. The antioxidants like epicatechin and ferulic acid have shown similar mechanisms as UA, in terms of suppressing the NF-κB activity in gamma irradiated mice, and further reducing the oxidative stress (Sinha et al., 2012; Das et al., 2014).

In summary, UA, as an antioxidant, protects against cell death, and inhibits radiation-induced free radical production, lipid peroxidation, oxidative DNA damage, inflammation and NF-κB pathway activation. Moreover, the treatment of UA can prolong survival in mice exposed to whole body gamma irradiation, exert protective effects on the hematopoietic system, reduce excessive inflammatory responses, and reverse the increased expression of p65 and phospho-p65 induced by gamma irradiation. Given its radioprotective efficacy, low toxicity, and pharmaceutical properties, UA may represent a potential novel agent in protecting against gamma irradiation-induced deleterious effects.

## Author Contributions

Conceived and designed the experiments: M-KS, GS, HW. Performed the experiments: WH. Wrote and edited the paper: HW, WL, AC, SA, FT, and GS.

## Conflict of Interest Statement

The authors declare that the research was conducted in the absence of any commercial or financial relationships that could be construed as a potential conflict of interest.

## Supplementary Material

FIGURE S1 BJ human skin fibroblast cell viability after 20 Gy gamma radiation. UA + 20 Gy and 20 Gy group cells were treated with or without UA at 10 μM 16 h before exposure to 20 Gy gamma radiation. EMEM group without any drug treatment and gamma radiation exposure served as control. The cell viability was evaluated by MTT 24 h post-exposure. All data were expressed as mean ±SEM. Comparisons between groups were analyzed by One-Way ANOVA followed by Tukey’s *post hoc* test. ^∗^*p* < 0.05 vs. non-irradiated. ^#^*p* < 0.05 vs. 20 Gy.

FIGURE S2 Effects of UA on free radical production in BJ human skin fibroblast cells after gamma radiation. UA + 20 Gy and 20 Gy group cells were treated with or without UA at 10 μM 16 h before exposure to 20 Gy gamma radiation. EMEM group without any drug treatment and gamma radiation exposure served as control. ROS production was assayed by 5 μM DHE, and then subjected to the fluorescent reading. All data were expressed as mean ±SEM. Comparisons between groups were analyzed by One-Way ANOVA followed by Tukey’s *post hoc* test. ^∗^*p* < 0.05 vs. non-irradiated. ^#^*p* < 0.05 vs. 20 Gy.

FIGURE S3 Effects of UA on lipid peroxidation **(A)** and oxidative DNA damage **(B)** in BJ human skin fibroblast cells after gamma radiation. UA + 20 Gy and 20 Gy group cells were treated with or without UA at 10 μM 16 h before exposure to 20 Gy gamma radiation. EMEM group without any drug treatment and gamma radiation exposure served as control. **(A)** Lipid peroxidation in BJ human skin fibroblast cells was assayed by 50 μM DPPP. Fluorescence was read using excitation and emission wavelengths of 351 and 380 nm, respectively. **(B)** Oxidative DNA damage was evaluated by 8-OH-dG EIA kit. The absorbance at a wavelength of 420 nm was recorded and used to calculate the 8-OH-dG concentration based upon the standard curve. All data were expressed as mean ±SEM. Comparisons between groups were analyzed by One-Way ANOVA followed by Tukey’s post hoc test. ^∗^*p* < 0.05 vs. non-irradiated. ^#^*p* < 0.05 vs. 20 Gy.

FIGURE S4 Effects of UA on gamma irradiation-induced NF-κB activation. UA + 20 Gy and 20 Gy group cells were treated with or without UA at 10 μM 16 h before exposure to 20 Gy gamma radiation. EMEM group without any drug treatment and gamma radiation exposure served as control. **(A)** NF-κB DNA binding assay was performed 24 h post-exposure. **(B,C)** Phosphorylation and translocation of NF-κB were evaluated by western blot analysis. Representative image of western blotting **(B)** and densitometric analysis **(C)** of the expression of p65, phospho-p65, PARP, phospho-lκBα, IκBα, and β-actin. NE, nuclear extract. CE, cytoplasmic extract. Data were expressed as mean ±SEM. Comparisons between groups were analyzed by One-Way ANOVA followed by Tukey’s *post hoc* test. ^∗^*p* < 0.05 vs. non-irradiated. ^#^*p* < 0.05 vs. 20 Gy.

## Abbreviations

8-OH-dG: 8-hydroxy-2-deoxy Guanosine
BSA: bovine serum albumin
DCFDA: 2′,7′-Dichlorofluorescin diacetate
DHE: dihydroethidium
DIRP: Defence Innovative Research Programme
DMEM: Dulbecco’s Modified Eagle Medium
DMSO: dimethyl sulfoxide
DPPP: diphenyl-1-pyrenylphosphine
DPPP-O: diphenyl-1-pyrenylphosphine oxide
ECL: enhanced chemiluminescence
ELISA: enzyme-linked immunosorbent assay
EMEM: Eagle’s Minimum Essential Medium
FBS: fetal bovine serum
HRP: horseradish peroxidase
IACUC: Institutional Animal Care and Use Committee
IL-1β: interleukin-1beta
IL-6: interleukin-6
ROS: reactive oxygen species
MTT: 3-(4,5-dimethylthiazol-2-yl)-2,5-diphenyltetrazolium bromide
SDS: sodium dodecyl sulfate
TNF-α: tumor necrosis factor alpha
UA: ursolic acid
UAL: ursolic acid liposome
