# Supplementary figures and images for: Potential Protective Effects of Ursolic Acid against Gamma Irradiation-Induced Damage Are Mediated through the Modulation of Diverse Inflammatory Mediators
Source: Front Pharmacol. 2017 Jun 16;8:352. doi: 10.3389/fphar.2017.00352 (PMC5472704; doi:10.3389/fphar.2017.00352)

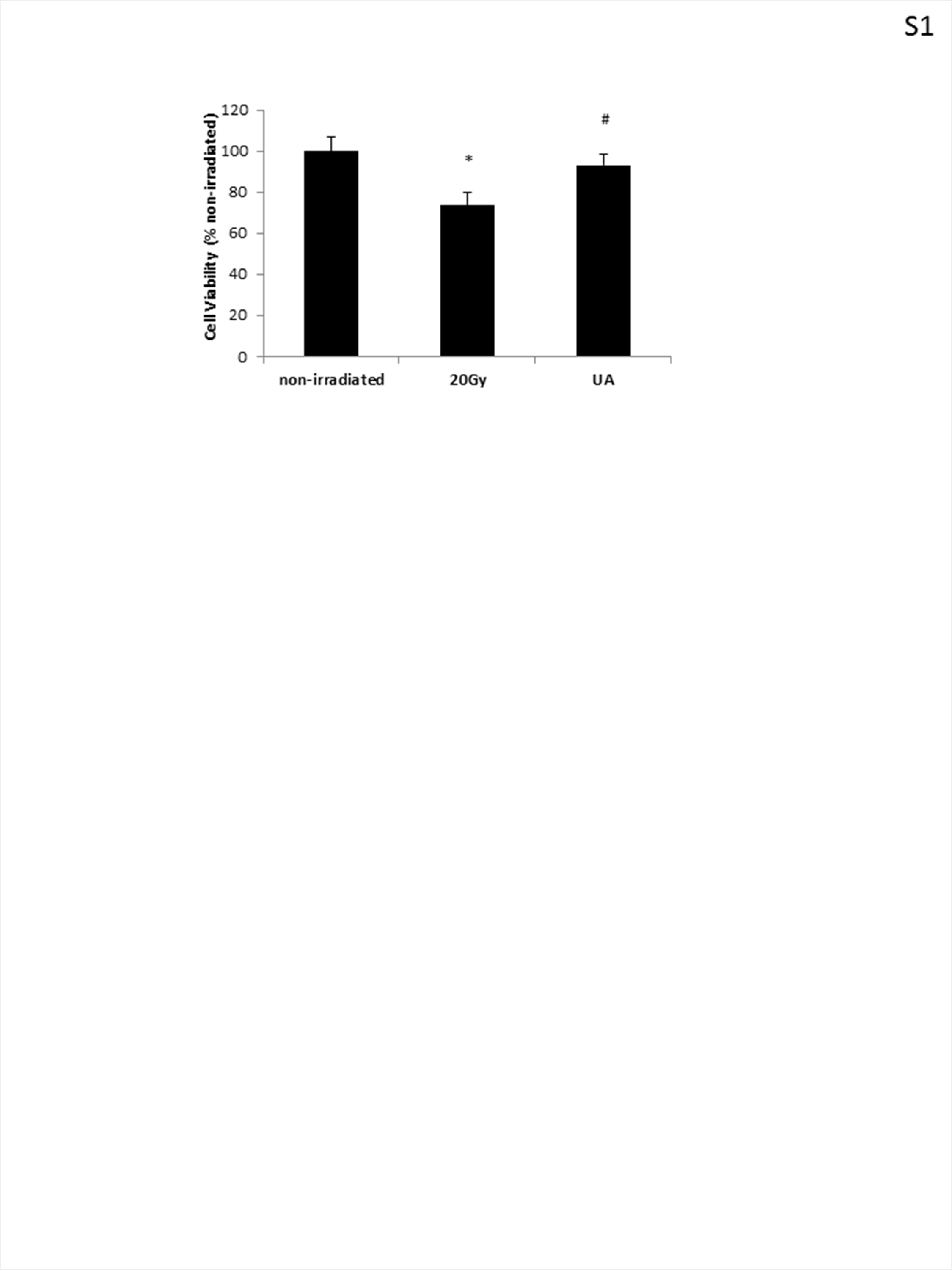

Supplement: FIGURE S1 — BJ human skin fibroblast cell viability after 20 Gy gamma radiation. UA + 20 Gy and 20 Gy group cells were treated with or without UA at 10 μM 16 h before exposure to 20 Gy gamma radiation. EMEM group without any drug treatment and gamma radiation exposure served as control. The cell viability was evaluated by MTT 24 h post-exposure. All data were expressed as mean ±SEM. Comparisons between groups were analyzed by One-Way ANOVA followed by Tukey’s post hoc test. ∗p < 0.05 vs. non-irradiated. #p < 0.05 vs. 20 Gy. [file Image_1.TIF]

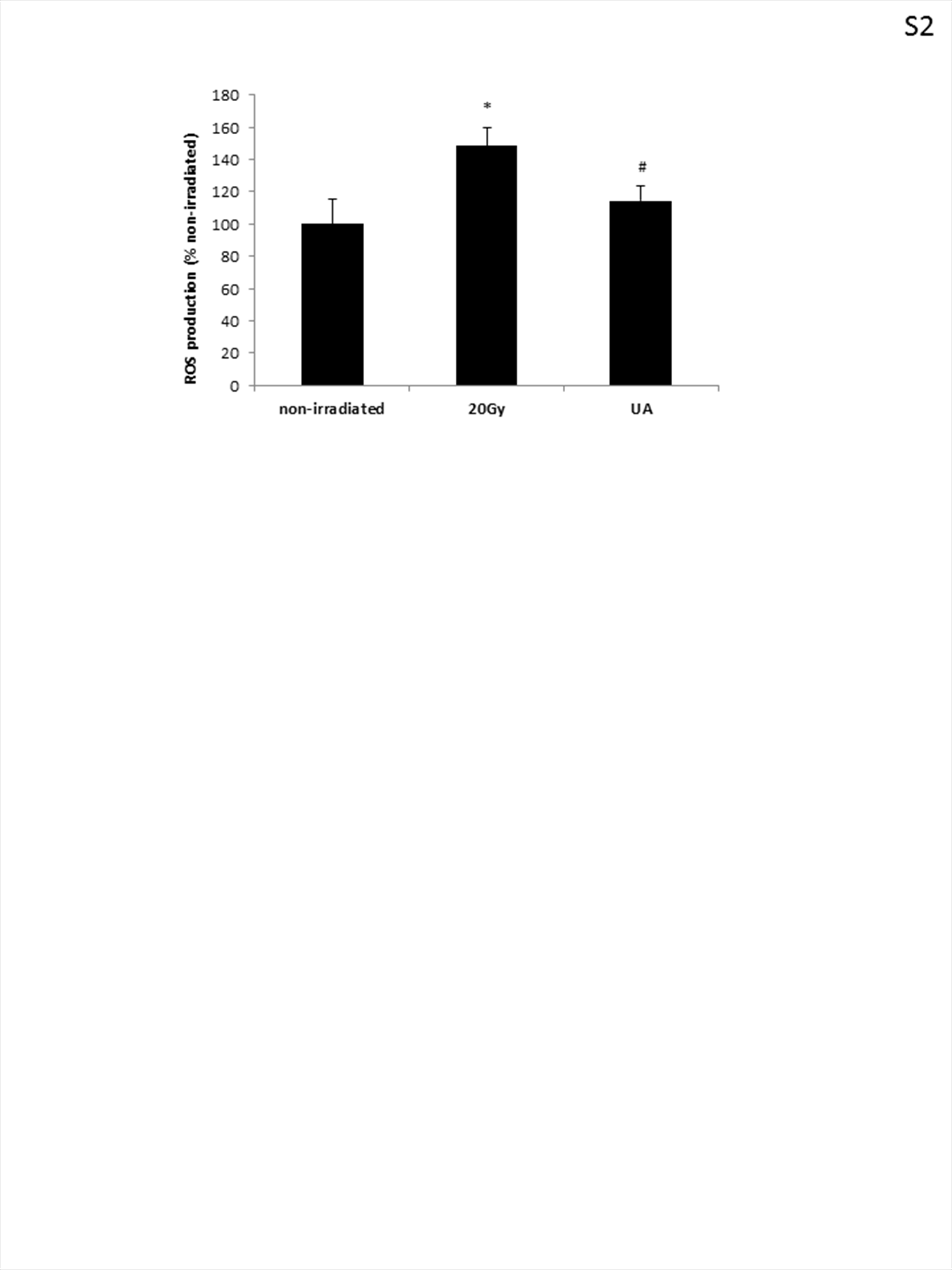

Supplement: FIGURE S2 — Effects of UA on free radical production in BJ human skin fibroblast cells after gamma radiation. UA + 20 Gy and 20 Gy group cells were treated with or without UA at 10 μM 16 h before exposure to 20 Gy gamma radiation. EMEM group without any drug treatment and gamma radiation exposure served as control. ROS production was assayed by 5 μM DHE, and then subjected to the fluorescent reading. All data were expressed as mean ±SEM. Comparisons between groups were analyzed by One-Way ANOVA followed by Tukey’s post hoc test. ∗p < 0.05 vs. non-irradiated. #p < 0.05 vs. 20 Gy. [file Image_2.TIF]

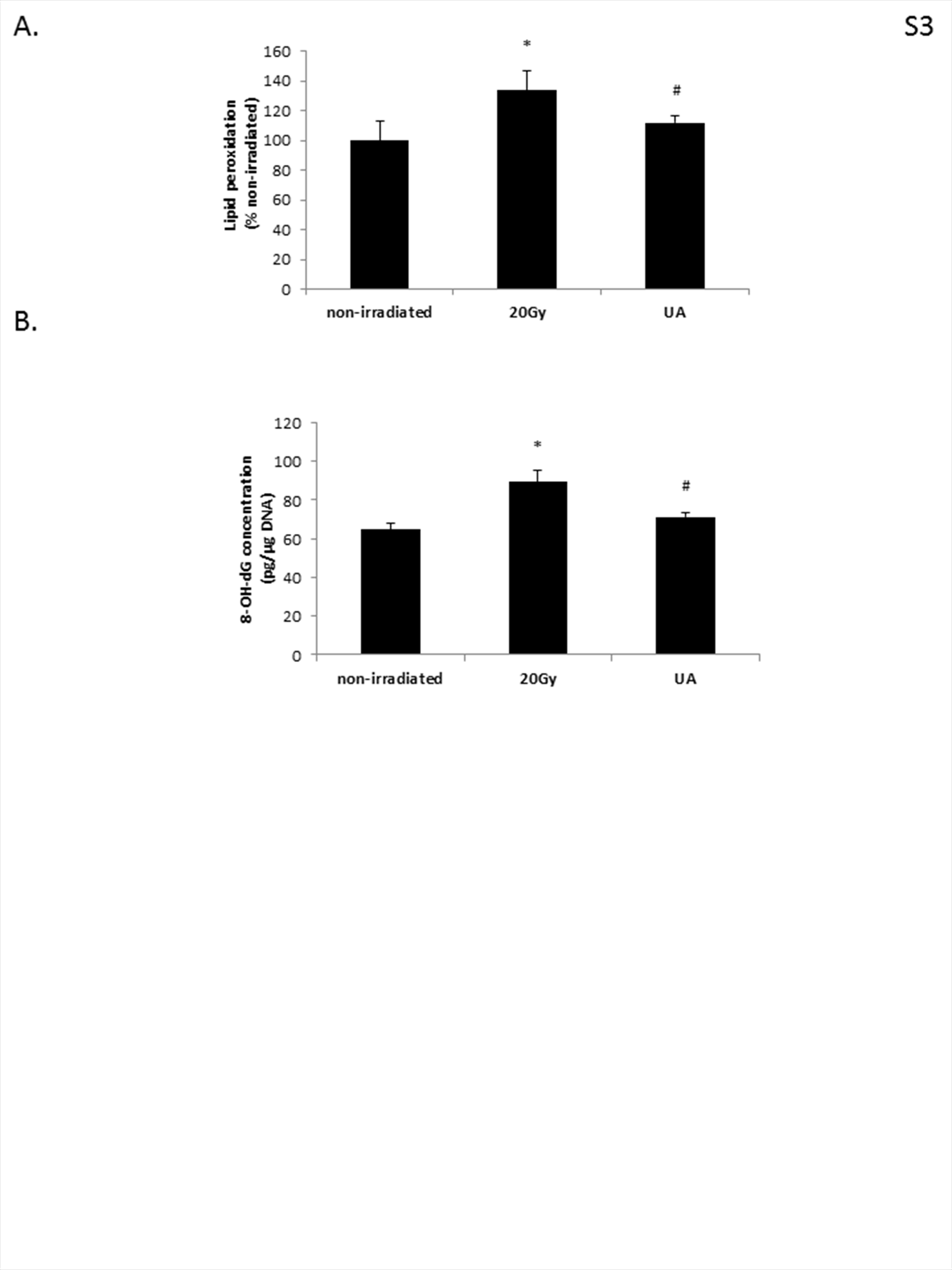

Supplement: FIGURE S3 — Effects of UA on lipid peroxidation (A) and oxidative DNA damage (B) in BJ human skin fibroblast cells after gamma radiation. UA + 20 Gy and 20 Gy group cells were treated with or without UA at 10 μM 16 h before exposure to 20 Gy gamma radiation. EMEM group without any drug treatment and gamma radiation exposure served as control. (A) Lipid peroxidation in BJ human skin fibroblast cells was assayed by 50 μM DPPP. Fluorescence was read using excitation and emission wavelengths of 351 and 380 nm, respectively. (B) Oxidative DNA damage was evaluated by 8-OH-dG EIA kit. The absorbance at a wavelength of 420 nm was recorded and used to calculate the 8-OH-dG concentration based upon the standard curve. All data were expressed as mean ±SEM. Comparisons between groups were analyzed by One-Way ANOVA followed by Tukey’s post hoc test. ∗p < 0.05 vs. non-irradiated. #p < 0.05 vs. 20 Gy. [file Image_3.TIF]

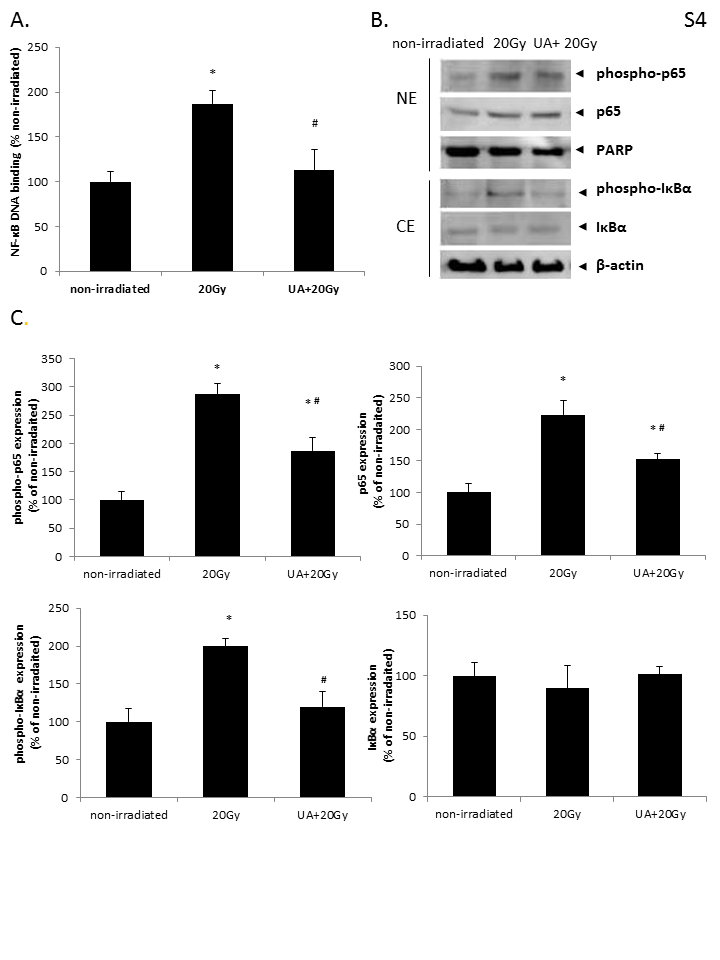

Supplement: FIGURE S4 — Effects of UA on gamma irradiation-induced NF-κB activation. UA + 20 Gy and 20 Gy group cells were treated with or without UA at 10 μM 16 h before exposure to 20 Gy gamma radiation. EMEM group without any drug treatment and gamma radiation exposure served as control. (A) NF-κB DNA binding assay was performed 24 h post-exposure. (B,C) Phosphorylation and translocation of NF-κB were evaluated by western blot analysis. Representative image of western blotting (B) and densitometric analysis (C) of the expression of p65, phospho-p65, PARP, phospho-lκBα, IκBα, and β-actin. NE, nuclear extract. CE, cytoplasmic extract. Data were expressed as mean ±SEM. Comparisons between groups were analyzed by One-Way ANOVA followed by Tukey’s post hoc test. ∗p < 0.05 vs. non-irradiated. #p < 0.05 vs. 20 Gy. [file Image_4.TIF]
